# Exploring human-robot cooperation with gamified user training: a user study on cooperative lifting

**DOI:** 10.3389/frobt.2023.1290104

**Published:** 2024-01-12

**Authors:** Gizem Ateş Venås, Martin Fodstad Stølen, Erik Kyrkjebø

**Affiliations:** Department of Computer Science, Electrical Engineering and Mathematical Sciences, Førde, Norway

**Keywords:** HRC, IMU, human motion tracking, co-lift, user training, gamification

## Abstract

Human-robot cooperation (HRC) is becoming increasingly relevant with the surge in collaborative robots (cobots) for industrial applications. Examples of humans and robots cooperating actively on the same workpiece can be found in research labs around the world, but industrial applications are still mostly limited to robots and humans taking turns. In this paper, we use a cooperative lifting task (co-lift) as a case study to explore how well this task can be learned within a limited time, and how background factors of users may impact learning. The experimental study included 32 healthy adults from 20 to 54 years who performed a co-lift with a collaborative robot. The physical setup is designed as a gamified user training system as research has validated that gamification is an effective methodology for user training. Human motions and gestures were measured using Inertial Measurement Unit (IMU) sensors and used to interact with the robot across three role distributions: human as the leader, robot as the leader, and shared leadership. We find that regardless of age, gender, job category, gaming background, and familiarity with robots, the learning curve of all users showed a satisfactory progression and that all users could achieve successful cooperation with the robot on the co-lift task after seven or fewer trials. The data indicates that some of the background factors of the users such as occupation, past gaming habits, *etc.*, may affect learning outcomes, which will be explored further in future experiments. Overall, the results indicate that the potential of the adoption of HRC in the industry is promising for a diverse set of users after a relatively short training process.

## 1 Introduction

Robotics has been a game changer in mass manufacturing by allowing various processes to be automated to produce a large number of items with the same quality, and often with a significantly shorter production time. For small and mid-size enterprises (SMEs) with smaller production volumes, the benefits of introducing industrial robots into production lines have not been as apparent and many have been reluctant to automate production processes (Fortune Business Insight, 2022). The introduction of collaborative robots (cobots) that can work next to human workers in the factory without fences has opened up a new potential in automation. However, robots and humans are still only taking turns when working on products, and the potential for Human-Robot Cooperation (HRC) where humans and robots can cooperate to work on the same product simultaneously has not been adopted by the industry (Michaelis et al., 2020).

### 1.1 Background

A HRC system aims to combine the superior skills of humans (problem-solving, decision-making *etc.*) and robots (precision, accuracy, repeatability, *etc.*) to accomplish the task more efficiently and accurately. There are several factors in HRC usability such as safety, trust, user’s experience, effectiveness, efficiency, learnability, flexibility, robustness and utility (Lindblom et al., 2020; Simone et al., 2022). Although safety is the most important factor, it is not sufficient for achieving optimal usability from an HRC system. This study focuses on three integral components of HRC system usability: 1) a robust communication method characterized by reliability, adequate precision, and accuracy; 2) dynamic role allocation between the human and robot within the HRC framework; and 3) human operator proficiency in utilizing the system.

A human-robot team must have a reliable communication method to achieve successful HRC. The human input can be via joystick, voice, haptic and/or motion commands. The robot actions should be predictable and easily understood by human operators to ensure safe, intuitive and effective cooperation and build trust (Lee and See, 2004; Lindblom and Wang, 2018). Moreover, it is not straightforward to assign a leader to a human-robot team to achieve the task in the most optimal way. The robot and the human excel in different skills. Therefore, either their leadership roles should be allocated in a flexible manner depending on the task requirement/state, or prescribed optimally beforehand, to maximize system performance regardless of dynamic or static role assignment (Mörtl et al., 2012). Lastly, the human operator should have enough competence and training to understand system capabilities and usage and to handle system drawbacks and failures. In order for SMEs to benefit from using cobots for industrial applications, it is crucial that human operators receive proper training in using HRC systems (Michaelis et al., 2020).

User training is perhaps not adequately addressed in the context of HRC although the research has been going on within the HRC field for a couple of decades (Jost et al., 2020). The implementation of new technology in the industry has always been challenging for both employees and employers. Training is important not only to facilitate learning about how to use new technology but also to manage employee perceptions and attitudes about the new technology (Huber and Watson, 2013). Two major reasons for this challenge addressed in the literature are perceptions of the technology’s ease of use and perceptions of the technology’s usefulness (Marler et al., 2006). The importance of user training in Human-Robot Interaction (HRI) is addressed in Werner et al. (2020) on 25 elderly people who get help from a bathing robot. Nonetheless, a systematic approach and the effect of user training within the HRC field is lacking.

A few studies suggested three methodological approaches for user training in HRI. One methodology is the development of adaptive human-machine interfaces (HMIs) for industrial machines and robots. This approach involves measuring the user’s capabilities, adapting the information presented in the HMI, and providing training to the user (Villani et al., 2017). By adapting the interface to the user’s needs and abilities, the cognitive workload can be reduced, and the user can interact more effectively with the robot. The Wizard-of-Oz (WoZ) technique is another methodology used in human-robot interaction. In this technique, a remote supervisor drives the robot using a control interface to simulate an artificially intelligent robot (Tennent et al., 2018). Another approach is to use virtual/physical simulators mostly due to safety reasons (Mitchell et al., 2020), yet it increases the overall development cost of a novel HRC system. The current examples found in the literature are limited to mostly medical, surgical and military applications (Prasov, 2012; Dubin et al., 2017; Azadi et al., 2021) and the main training purpose is to train the user for the specific task rather than the user learning the HRC system itself.

Serious games (SG), gamification methods and game-based learning (GBL) can be used to develop supplemental training materials that are interesting and interactive, making it simpler for learners to apply their newfound knowledge (Susi et al., 2007; Kleftodimos and Evangelidis, 2018; Anil Yasin and Abbas, 2021). Several studies merged gamification and simulation in user training within various fields (Wang et al., 2016; Checa and Bustillo, 2020). According to Kapp (2012), “a serious game is an experience created using game mechanics and game thinking to educate people in a specific content domain”. In serious games, learning, training, and other objectives come first rather than pure entertainment. The effectiveness of the training outcome can be increased by gamification techniques and game elements (Pesare et al., 2016). SG and GBL have been popularly used in various training purposes since 2013 (Checa and Bustillo, 2020) such as in fatigue assessment (Kanal et al., 2020), rehabilitation (Andrade et al., 2013), constructing better communication with individuals who has Autism Spectrum Disorder (Silva et al., 2018) *etc.* Although it is not common in user training in HRC applications, one successful study used a serious virtual reality game that simulated the cooperation between industrial robotic manipulators (Matsas and Vosniakos, 2017). There is still room for merging SG, GBL and gamification in user training in HRI and HRC (Jones et al., 2022).

**FIGURE 1:**
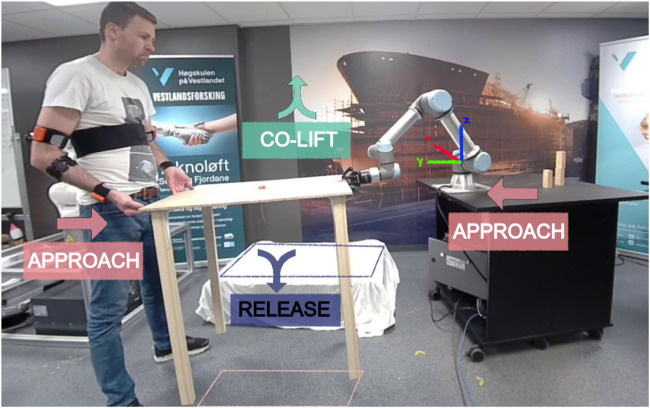
The full-cycle of co-lift task and its states (APPROACH, CO-LIFT and RELEASE) and the pick and place locations of the common object. The system uses IMUs for human motion estimation and an EMG-sensor to open/close the gripper (Ates et al., 2022).

Cooperative lifting (co-lift) scenario is a common example in HRC where humans and robots lift and carry heavy, flexible, or long objects together while exploiting human cognitive skills and robot accuracy in different parts of the task. The co-lift task was chosen as the experimental study scenario for HRC in this article due to the fact that material manipulation applications (e.g., handling, positioning, polishing) have been found to be the most common tasks in the industry with more than 20% of the total number of tasks (Parra et al., 2020). There are several studies on co-lift and manipulation between a human and a robot in the literature. In Mörtl et al. (2012), the authors used haptic data to dynamically assign the leader roles between the human and robot in a co-lift scenario. A recent study presented in Liu et al. (2021) estimated the external forces applied by the human operator during the collaborative assembly of a car engine. In Ramasubramanian and Papakostas (2021), the human operator and a collaborative robot on a mobile platform carried a long stick between two locations in the work environment. In Nemec et al. (2017), speed and disturbance rejection were adjusted for transporting an object through learning by demonstration. While these studies cover important topics for HRC and co-lift tasks, they present solutions only in the active carrying phase. It is important to address the before (approach) and after (release) phases of the active co-lift phase as shown in Figure 1 elaborating with the human input method so that the chain or repeated HRC tasks can automatically restart without any interrupts. Overall, co-lift is a relatively common task within HRC and versatile such that various aspects of user training can be observed in different phases of co-lift.

### 1.2 Research questions and outline

The HRC field is rapidly growing. We think that human-machine interaction should be considered as a whole when a new HRC system is developed. As a daily life example, driving a car is a relevant human-machine interaction. Individuals are required to undergo a comprehensive training process to obtain a driving license, which is crucial for ensuring the safe and effective operation of vehicles. This process might be apprehended as cumbersome for many and driving a car is perceived as complicated. However, if society had waited for cars to achieve perfection before embracing their use, disregarding the potential of user training, our progress as a civilization would have been severely hindered.

Our motivation in this study is to gauge people’s capability to cooperate with robots and observe the effect of user training on various demographics. The objective is to conduct a quantifiable empirical unbiased observation on the quality of HRC and the effect of user training. We investigate the importance of user training in HRC and examine if a particular demographic group in terms of age, gender, occupation, familiarity with robots, gaming backgrounds, *etc.*, plays a fundamental role in cooperating with the robots and/or learning how to cooperate with a robot. It is suggested in the literature that the performance of HRC could be affected by the worker’s previous experience with robotics (Simone et al., 2022), their personality Walters et al. (2005) as well as their gaming background (Tanaka et al., 2016). Moreover, we aim to provide a systematic approach to develop a user training procedure for HRC using gamification. We explore the potential of including game elements rather than comparing gasified training to other training approaches.

The outline of this paper is as follows: the methodology of developing a novel human-motion-based HRC system using IMUs as the motion capturing system (Ates et al., 2022) in a co-lift scenario is in section 2.1 and section 2.2, the gamification procedure is in section 2.3, the setup for user experiments is in section 2.4, the results are in section 3 and the discussion of findings are in section 4.

Although the results are solely dependent on the specific type of application, these findings should be considered for the evaluation of the effective convenience of the cobots, including an analysis of the variation in the workers’ performance, and consequently, of the entire HRC system.

## 2 Materials and methods

### 2.1 Cooperative lifting

**FIGURE 2:**
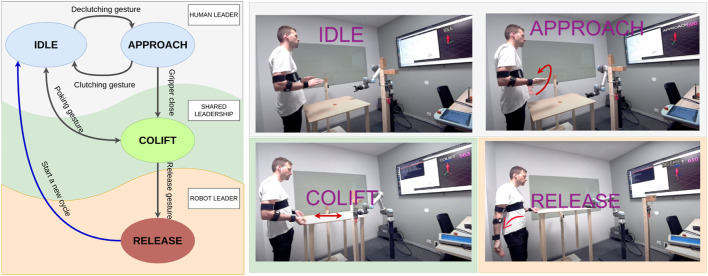
HRC roles and states in the experimental co-lift scenario. Human Leading role has two states: IDLE: Both chest-to-wrist (hand) motions are actively calculated yet no motion command is sent to the robot. APPROACH: Combined (merged) pose from two hands is actively calculated to produce soft real-time end-effector goal pose commands. The robot follows the human actively. Shared Leadership role has one state: CO-LIFT: The robot applies a directional compliant force and the human decides the direction of this force with elbow gestures. Robot Leading role has one state: RELEASE: The robot takes the lead in the operation, and moves to a predefined release pose. The human follows the robot’s motions. The steering arm is assigned for transition gestures and the red arrows show the respective transition gestures between states. Rotating the right palm up/down is assigned as the de-clutching/clutching gesture, clenching the right fist is the gripper close gesture (not highlighted in the fig.), pushing/pulling the table is the poking gesture, and releasing the right arm down is the release gesture.

The operation is divided into different states, and the roles of the human and robot change throughout the states as shown in Figure 2. The system starts in the IDLE state, where no motion is transferred from the human to the robot. The human can then use a clutching gesture to transit between IDLE and APPROACH states, where motion control commands are sent to the robot based on the pose information of the human’s hands. In the CO-LIFT state, the human and robot share leadership and perform complex motions specific to the application scenario. The human can then gesture the robot to enter the RELEASE state, where the robot takes charge of the position and velocity control of the object to place it accurately in a predefined position.

### 2.2 Human motions to robot actions

Studies on human motion tracking and estimation can be categorized based on the type of motion tracker devices used: visual-based (Morato et al., 2014; Sheng et al., 2015), and nonvisual-based (Roetenberg et al., 2009; Kok et al., 2014), and hybrid solutions (Sugiyama and Miura, 2009). The visual-based solutions are widespread in motion tracking since they provide highly accurate human motion tracking but often fail in industrial usage due to occlusion, loss in line-of-sight, intolerant to lightning changes, and lack of mobility (Rodríguez-Guerra et al., 2021). Common alternatives to non-visual systems are IMU-based solutions which are stand-alone systems without no permanent installations. They often cost considerably less than their visual alternatives but are prone to drift for long-term usage. While several solutions to eliminate the drift problem have been proposed (El-Gohary and McNames, 2015; Kok et al., 2017; Ludwig and Burnham, 2018), there are still a few examples using IMU-based solutions particularly in real-time in HRC applications. Although IMUs are selected as the main motion tracking devices, the selected human motion tracking technology is not the most critical point in this study.

#### 2.2.1 Human motion estimation (HME)

**FIGURE 3:**
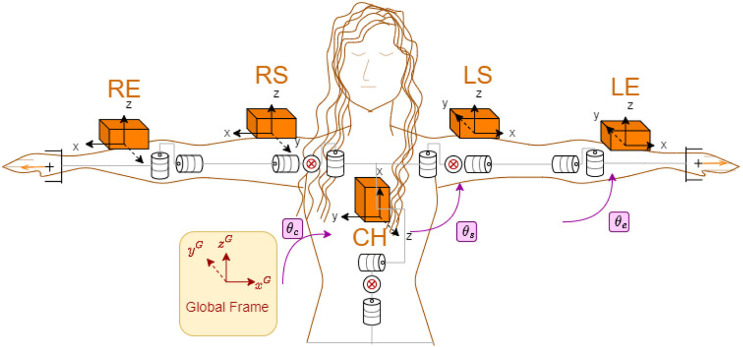
Human model and IMU placement on right/left elbow (RE/LE), right/left shoulder (RS/LS) and chest (CH) (Ates et al., 2022).

In this study, we used 5 IMUs as the motion-tracking system. After acquiring the 3D orientations from individual IMUs, the biomechanical model of the human body is placed in the calculations. In order to measure the full upper-body motions we placed the IMUs as shown in Figure 3. Nonetheless, the selected motion capture technology is not critical and any type of human-motion estimation method would work as long as the human input is appropriate to create a real-time goal pose command for the robot.

With this model, we measure 13 degrees of freedom (DoF) upper-body motions including the chest, upper and lower arm motions on both arms but neglected the wrist motions. Our human model is a collection of estimated individual joint angles, where a joint angle can be found by calculating the rotation between two consecutive links with attached IMUs. The kinematic chain for such a human model from the base (chest) to the tip (left hand) can be written as:

$$q_{c}=q_{CH}^{GS}\;q_{s}=q_{c}^{*}⊗q_{LS}^{GS}\;q_{e}=q_{c}^{*}⊗q_{s}^{*}⊗q_{LE}^{GS}$$ (1)

where *q*_*c*_, *q*_*s*_ and *q*_*e*_ are the quaternions representing joint angle rotations, $q_{CH}^{GS}$ , $q_{LS}^{GS}$ and $q_{LE}^{GS}$ are the IMU orientation from global to the sensor’s frame which are the raw orientation readings from the IMUs. The same procedure is applied to the right arm. The lower body is taken as the fixed reference frame.

#### 2.2.2 Human biomechanical model

In human motion and gesture estimation, the first step is to define the human model. This model can be a silhouette as in Bradski and Davis (2000) or a biomechanical model as in Roetenberg et al. (2009); Cerveri et al. (2005). Since the human body contains more complex joints and links than ordinary actuators and link elements, it is not possible to model the human body with 100% accuracy. As a result of that, the total degrees of freedom (DoF) of the human model is not exact. For example, the human arm is modeled as 4 DoF in Theofanidis et al. (2016), 9 DoF in Phan et al. (2017) and 7 DoF in Ghosal (2018).

#### 2.2.3 Human to robot motion translation

Human-to-robot motion translation converts estimated human motions and gestures into the desired goal pose for the robot manipulator in real-time. The human arm motion is not directly mimicked by the robot. Depending on the states explained in more detail in Section 2.1, different human-to-robot motion mapping methods are applied. This subsection presents how a robot manipulator goal pose command is created based on given human arm motions. The human arm is modelled as 5 DoF and the robot used in this study is a 6 DoF (RRRRRR) manipulator with a spherical wrist configuration.

In the case of active command cooperation, two arm motions of the human are merged to create one end-effector goal pose. In the case of passive command cooperation, two elbow poses are used.

##### 2.2.3.1 Active command cooperation scenario

The relative motions of both human arms are merged and translated into a single goal pose for the robot as introduced in Ateş and Kyrkjebø (2021). The merged hands pose is calculated based on the relative motions of each arm. We refer to the arms as the motion arm and steering arm. The steering arm is also responsible for clutching and state transitions. In our setup, the steering arm is the right arm but this can be changed in the merged hand pose equation (Eq. 2).

$${\hat{P}}_{h,t}^{-}=\hat{s}\cdot \left({\hat{P}}_{hm,t=0}^{-1}\times {\hat{P}}_{hm,t}\right)+\hat{k}\cdot \left({\hat{P}}_{hs,t=0}^{-1}\times {\hat{P}}_{hs,t}\right)$$ (2)

where ${\hat{P}}_{h,t}^{-}$ is the merged hand pose at *t* = 0^−^, ${\hat{P}}_{hm,t=0}$ is the motion hand’s pose, ${\hat{P}}_{hs,t=0}$ is the steering hand’s pose. The multiplication with their inverse at *t* = 0 simply sets the pose readings to zero for relative motion mapping.

Also, different weights for each arm motion can be defined by the scaling factors $\hat{s}$ and $\hat{k}$ in Eq. 2 in the code but for these experiments, they both set to the same multiplier.

The robot goal pose based on the merged hand pose is

$$\hat{H}\left(t\right)={\hat{P}}_{h,t=0}^{-1}\times {\hat{P}}_{h,t}\;{\hat{P}}_{r,t}={\hat{P}}_{r,t=0}\times \hat{H}\left(t\right)$$ (3)

where $\hat{H}\left(t\right)$ is the transformation of the merged hand pose from the initial to the current pose.

##### 2.2.3.2 Predefined command cooperation scenario

**FIGURE 4:**
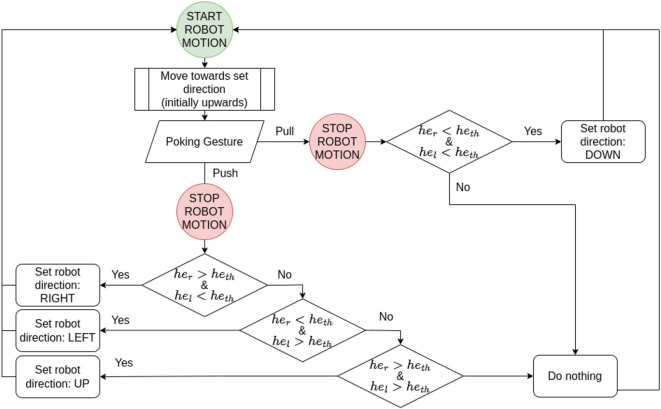
Predefined command cooperation in the active lifting phase (i.e., COLIFT state).

When the human and the robot are both in the leadership role (i.e., carrying the object together, defined as COLIFT state in Sect.2.1) the predefined command cooperation method is applied. There the human uses elbows to show the direction where the robot should go with a poking gesture (i.e., by pulling/pushing the object). The motion type is predefined such that the robot goes upwards, downwards, left and right in the xz-plane. The flowchart of how the predefined command cooperation in the active lifting phase is implemented is given in Figure 4.

### 2.3 Gamification

Gamification methods in HRC user training are explained in detail in our previous study (Ates and Kyrkjebo, 2022). The time constraint is directly connected to the score element. The game (i.e., each trial) is supposed to be finished in 10 min. The user starts with 600 points and loses 1 point every second. There are 2 buttons within the common workspace that represent physical waypoints and are defined as achievement elements. Each successful button press gives an additional 60 points. The game elements used in this study and their role is listed below.

• Themes create interest and engagement in educational games. It could be some sort of introductory backstory or a narrative that accompanies the entire game. For the proposed HRC scenario, the background story is presented as the research objectives.
• Achievements are the mechanisms connecting the target outcomes of the HRC task that the gained user skills.
• The game score is calculated based on the user’s performance, which only depends on how quickly they complete each achievement in this study. Additional parameters can be enabled such as the measurements taken from the IMU on the carried table regarding the tilting, deviation and trembling of the table.
• Challenges and conflicts are central elements in any game and they can be physical obstacles, battles with other players, or puzzles that need to be solved. In this study, the physical targets are set to be challenging elements.
• In an entertainment game, feedback is usually immediate and continuous which can be delivered through, reward points, additional ‘powers’ within the game or direct messages. In this study, both immediate and direct message feedback types are used through three main elements: simulated human model, game score and HRC state via the visual feedback unit. No *guided* feedback is implemented but the procedure was supervised by a human expert and objective guidance to all users is provided as needed. The objectivity in guidance is explained in section 2.4.3.
• Time constraint is a particular type of challenge in serious games, which is a powerful tool to push user limits. In this study, the time constraint is directly connected to the score element.
• Self-expressions are the parameters and preferences of the game which are up to the user. The user could ask to change the speed and the responsiveness of the robot. Moreover, in the proposed HRC case, the initial poses of the user’s hands, and the way the user grabs the table are not restricted but only guided before the trials. These are open questions in the relevant HRC study and user self-expressions are believed to be helpful in deciding the most optimal usage.
• Aesthetics are the game’s visual, aural and artistic elements in a gasified system. They play an engaging and immersive role in the game. In an HRC scenario, the real robot can be perceived as an aesthetic element. Nonetheless, it is still important to add aesthetic elements to help the user engage in the task. In this study, the physical buttons incorporate LEDs that are activated when the button is pressed. Additionally, a graphical user interface is developed for the procedures that the user is able to see throughout the experiment.

### 2.4 User experiments

This section explains how the cooperative lifting operation is set up for user experiments using human motion and gesture information, and gaming elements. The technical setup is explained in detail, along with the user recruitment and selection procedure, the experimental procedure that all participants perform in the experiments, and the learning criteria users are evaluated by. The experimental procedure was carried out in 4 stages: pre-survey, video tutorial, physical human-robot experiment, and post-survey.

#### 2.4.1 Technical setup

**FIGURE 5:**
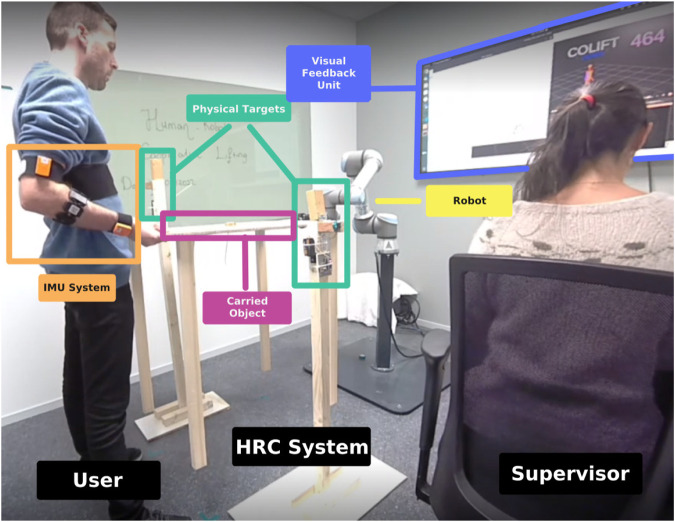
Experiment room and the units of the training system.

The majority of the physical experimental part is suggested in our previous study (Ates and Kyrkjebo, 2022). The human-robot experimental system was set up in ROS master PC which is a standard laptop with ROS Noetic installed. The ROS master handles all communication between the Xsens Awinda wireless IMU system, physical buttons connected to Arduino Uno microcontrollers transmitting wirelessly via NRF24L01+ RF transmitters, and the universal Robot UR5e cobot which is connected to the same local network as shown Figure 5.

#### 2.4.2 User recruitment and selection

The user experiments took place at Western Norway University of Applied Sciences, Campus Førde in autumn 2022. The announcements for recruitment to the experiment were distributed through social media, flyers at the University, and through the local newspaper. A total of 40 healthy participants between ages of 20–54 were recruited in total, 8 of which were used as pilot users to optimize experimental parameters such as the number of trials, the robot interaction force threshold in the co-lift state, human-robot directions (mirrored mapping), and some additions to the GUI. Data from the pilot experiments are not included in the paper. In total, 32 participants did ten or more trials on the human-robot colift system, and were assigned a 5-digit random ID and stored anonymously according to Norwegian Centre for Research Data standards^1^. Users with severe physical disabilities were excluded from participating in the experiments.

#### 2.4.3 Pre- and post-survey

All participants were asked to fill in a pre-survey and a post-survey form online using Microsoft Forms. In the pre-survey, users were asked general personal questions, related to interest in robotics and familiarity with robots. In the post-survey, the users were asked to asses several elements of the system such as the visual feedback, leadership roles between them and the robot, difficulty of the task in the four states, intuitiveness of the system and fatigue. The answers are collected either as short texts or a selection from a Likert scale depending on the question.

User’s heights and arm lengths are measured before the physical experiment. The user was asked to keep the shoes on during the height measurement since we are interested in the effective height the user while s/he was performing the task. Arm length measurement was the length between right wrist to right shoulder origin.

After the pre-survey, a tutorial video^2^ was made available to users to show them the task and how the system works. The video was uploaded on Youtube as *Unlisted* and the access link was only shared with the subjects before the physical experiments. To minimize the bias, users who had completed the experiment were asked not to discuss the details of the procedure with other participants. To ensure objective guidance, if during the experiment, the participant asks a question whose answer is already given in the video, then the supervisor provides an answer. If the question is something which is not addressed in the video or consent form, then the supervisor denies replying to such questions. After the physical experimental procedure, the post-survey was applied.

To analyse if any background factors played a role in how well users can learn to cooperate with robots, we divided participants into two samples based on the survey responses. For the gender and job category, the samples were *Woman - Man* and *IT/Engineering - Other*. For other categories, samples were divided into *Low - High* based on the numerical selection on the scale in the respective survey question. For the majority of the categories, the integer mid-point of the Likert scale was selected as the threshold for dividing into samples. However, for some questions, the participants’ answers were weighed on only one side of the threshold such as for physical tiredness and mental tiredness, and we used the mean of the answers instead of dividing participants into two samples.

We applied a two-tailed *t*-test for each category with respect to each learning criterion. The null hypothesis was the same for all categories: *“There is no difference between sample-* X *and sample-* Y *in learning criteria* L ”. We analyzed the samples with respect to the learning criteria where the null hypothesis was rejected with 10% significance level.

The *t*-test formula *t* for unequal sizes and variances between samples and the degree of freedom calculation *df* in Eq. 4 was used.

$$t=\frac{\left(\bar{x}-\bar{y}\right)-\left(\mu_{x}-\mu_{y}\right)}{\sqrt{\frac{s_{x}^{2}}{n_{x}}+\frac{s_{y}^{2}}{n_{y}}}},df=\frac{{\left(\frac{s_{x}^{2}}{n_{x}}+\frac{s_{y}^{2}}{n_{y}}\right)}^{2}}{\frac{{\left(\frac{s_{x}^{2}}{n_{x}}\right)}^{2}}{n_{x}-1}+\frac{{\left(\frac{s_{y}^{2}}{n_{y}}\right)}^{2}}{n_{y}-1}}$$ (4)

where $\bar{x}$ , $\bar{y}$ are the calculated means, *s*_*x*_, *s*_*y*_ are standard deviations and *n*_*x*_, *n*_*y*_ are the size of the sample- X and sample- Y , respectively. The term (*μ*_*x*_ − *μ*_*y*_) is the difference of hypothetical means of two samples, which is zero in our case based on the null hypothesis (*H*_0_: *μ*_*x*_ = *μ*_*y*_). The denominator is the estimated standard deviation $\left(SE_{\bar{x}-\bar{y}}\right)$ of the distribution of differences between independent sample means for unequal variances.

To calculate the effect size, we used both Cohen’s d and Hedge’s g because of the difference in variance between samples. To decide which one to use for particular categories, we set a threshold such that the difference between two standard deviations was less than the minimum of the two standard deviations. Therefore, the effect size calculation was

$$\left\{\begin{array}{cc} d=\frac{\left(\bar{x}--\bar{y}\right)}{\sqrt{\frac{s_{x}^{2}+s_{y}^{2}}{2}}}\; & \text{if}\left|s_{x}-s_{y}\right|<\left|\left(\text{min}\left(s_{x},s_{y}\right)\right.\right|\\ g=\frac{\left(\bar{x}--\bar{y}\right)}{\sqrt{\frac{\left(\left(n_{x}-1\right)\cdot s_{x}^{2}+\left(n_{y}-1\right)\cdot s_{y}^{2}\right)}{n_{x}+n_{y}-2}}}\; & \text{otherwise.} \end{array}\right.$$ (5)

#### 2.4.4 Experimental procedure

For the experimental procedure, we designed a co-lift operation that deliberately challenged participants to cooperate with the robot to achieve the goal of the operation. In the experiments, the human and the robot would pick a table whose location was not known to the robot, move it via two goal posts whose locations were also unknown to the robot, and place the table on a predefined final position known to the robot. The participants were scored based on the elapsed time, if they reached the goal posts, and if the overall co-lift operation was successful. Every participant started at 600 points, and lost one point each second. They gained 60 points for each successful goal post requiring that participant and the robot could cooperatively lift and move the table to trigger the physical buttons. When the last goal post was successfully reached, the user could gesture that the system should enter into the RELEASE state, and the robot would take the lead in placing the table on the final position to end the experiment and the score countdown.

All users started with the same robot speed. In the APPROACH state the servoL parameters for the robot were selected as acceleration = 0.5 m/*s* ^2^, velocity = 0.3 m/*s*, blocking time = 0.002 s, lookahead time = 0.1 s, and gain = 300. In the COLIFT state, the forceMode parameters were dependent on the direction, and the compliant force *F* upwards was set to 3 ⋅ *F*, downwards to 0.5 ⋅ *F*, and sideways to 1.5 ⋅ *F*. The maximum allowed end-effector speed along the compliant axes was 0.8 m/*s* for horizontal directions and 0.5 m/*s* for vertical directions. For non-compliant axes, the maximum allowed deviation along any axis was 0.3 m/*s*. The angular compliance limits along all axes were set to 0.17 *rad*/*s*. In the RELEASE state, the moveL end-effector speed was set to 0.25 m/*s* with acceleration = 1.2 m/*s* ^2^ in asynchronous mode. After the user felt comfortable in using the system with the current parameters, s/he could ask to speed up the robot in selected directions.

Experiments took a place in a distraction-free room where only the experiment conductor (supervisor) and the participant were present during the experiments. The visual feedback unit and the robot were visible to the participant from the same perspective as shown in Figure 5.

#### 2.4.5 Learning evaluation

To evaluate learning, we compared user scores of 10 trials using 6 different criteria.

• Highest: The best score for the participant.
• Average 3 (*avg_3*): Average of 3 best scores.
• Average of last 5 (*avg_last_5*): Average of last 5 trials.
• Variation of highest 3 (*var_3*): Variation of 3 best scores.
• Deviation from baseline (*delta_learning*): Difference between average of first two trials (*avg_first_2*) and last 2 (*avg_last_2*) trials.
• Learned trial (*trial_no_avg_reached*): The trial number for which the participant reached the *avg_3* score.

To eliminate the chance effect, the 3 best scores of the participants were averaged in *avg_3*, and to evaluate the level of how well the participant had learned the cooperation, the average score of the last 5 trials were calculated in *avg_last_5*. The standard deviation of the highest 3 scores were calculated in *var_3* to analyze if a particular high score was due to luck, or a more consistent learned cooperation. The average of the first two trials *avg_first_2* were compared to the average of the last two trials *avg_last_2* to get a measure in *delta_learning* of how much the participants had improved during trials. How fast the participant gets used to the system was calculated in (*trial_no_avg_reached*). We avoided using a single criterion and/or single trial score to evaluate learning to obtain a broader perspective on user learning.

## 3 Results

A total of 32 healthy adults participated in the experimental study, with 10 female and 22 male participants between 20 and 54 years old (*μ* = 34.2 and *σ* = 9.6). The users had different occupational backgrounds (health, IT, management, craftsmanship, pedagogy, unemployed, *etc.*) at different levels (students, employees) and had different previous experiences from interacting with robots (both on a technical and a social level). The presentation of the results is divided into two aspects. First, the measures of the system based on the motion and gesture data collected during the experiments is presented in Section 3.1. Second, how the participants were able to learn to use the system with respect to the various background factors collected in the pre- and post-survey is presented in Section 3.2.

### 3.1 HRC system measures

**FIGURE 6:**
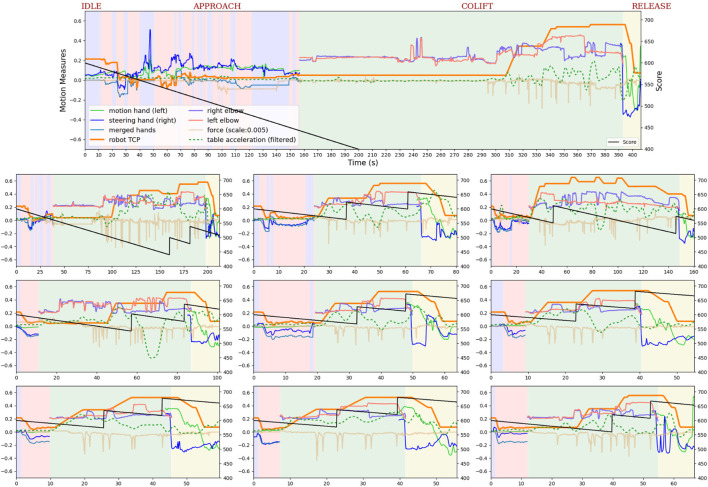
The figure shows the experimental measurements of user ID:35,764 during the 10 trials. The largest figure on the top is the first trial, and the rest of the trials are presented row by row from the top left to the bottom right. The experiments start with the IDLE state highlighted with purple background colour, followed by the APPROACH, COLIFT, and RELEASE states, which are highlighted with pink, green, and yellow background colours, respectively. The user’s two hand motions, calculated merged hands motion, and two elbow heights are measured from the participant’s side. The hand motions are visible shown in the IDLE, APPROACH and RELEASE states, and the elbow motions are shown only in the COLIFT state. The end-effector (TCP) motion and the exerted force are measured from the robot’s side, and the table acceleration and user score are measured as training parameters.

In total nine metrics related to the HRC system are presented in Figure 6. One participant (User ID:35,764) has been selected to illustrate the data from the experimental trials. The participant is a female health worker, more than 30 years old, has no current or past gaming habits, programming background and education in robotics, but stated that she has a cleaner robot at home/work in the user survey. The participant is not selected based on any particular background factor other than to visualize the data collected during the experiments. The user score data from all participants is provided for comparison as open access data for reproducibility (Venås, 2023)^3^.

From Figure 6, we see that the user score increases from the 1^*s*^ to the 10th trial. The duration of the first trial is about 400 s whereas the last is just over 60 s. There are quite a few transitions between the IDLE and the APPROACH states in the first trial.

Since the participant is not familiar with the system, she has a difficult time making the robot move in the desired direction and resets the hand motions several times by going back to the IDLE state. This type of several hand-reset behaviours is seen in trials 2, 3 and partly 4, but decreases as the participant learns/gets familiar with the system. From the 6^*th*^ trial the user reached a consistent level of performance.

Another point to highlight is the changes in the duration of the IDLE + APPROACH and COLIFT states. The duration of the IDLE + APPROACH state shows how comfortably the participant controls the robot’s motion using two-arm motions when the participant is the leader of the system. The first attempt to grab the table took around 160 s, whereas she managed to reduce it to under 10 s in later trials. The duration of the COLIFT state shows two things: 1) how comfortably the user controls the robot’s motion using instant gestures when the user cooperates with the robot, and 2) how well the grasping has succeeded. By default, the robot moves upward when the grasp on the table is complete. If the grasping is too abrupt, the robot will not automatically start moving upwards (as seen in trials 1, 2, 5 and 10) because it registers an initial poking gesture. The user needs to adjust the elbows’ poses adequately and poke the robot so that the initial upward motion starts again.

Some minor details to remark are the number of poking attempts during the COLIFT state, the magnitude of poking force, and the table acceleration over different trials. The table acceleration is pretty smooth overall for this user except for trial 5. In the first trials, there are significantly more poking attempts than in the last trials (except for trial 10). The poking force is well above the threshold which could be related to uncertainty about the system responsiveness. After the 6^*th*^ trial, there is more consistent poking behaviour. Note that the slight inconsistency in the 10th trial with regards to the upwards trend in the score and consistent poking behaviour is discussed in Section 4.1.

### 3.2 User learning evaluation

**FIGURE 7:**
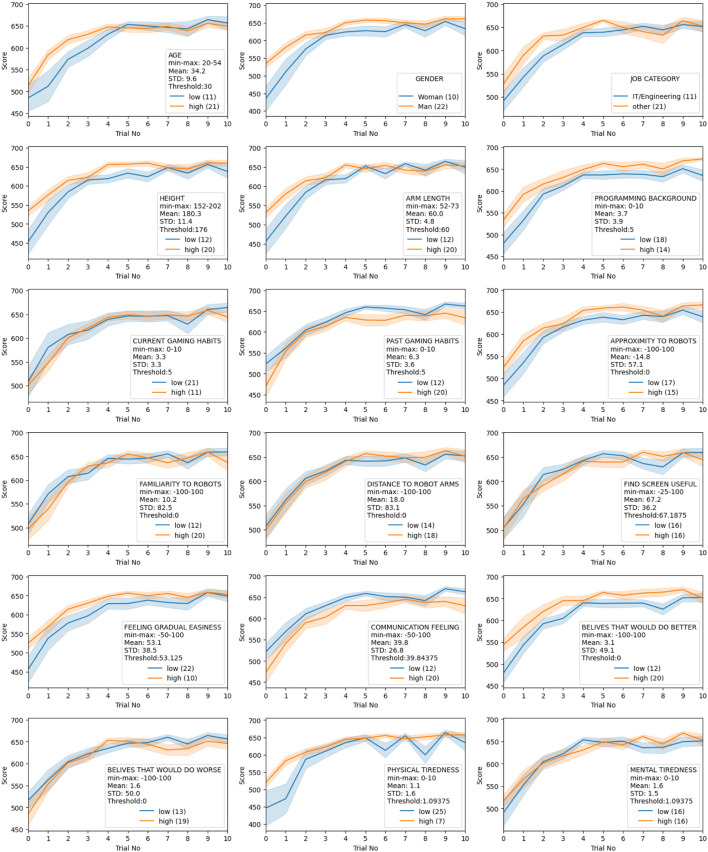
Participant background data on learning curves. Each plot shows the score of participants divided into two sample groups for the respective background variable. The plots show the aggregation of all participant scores in each sample group for each trial; the middle line shows the mean, and the shadow region shows the standard error.

We divided the user sample into two groups for 18 different user parameters such as age, gender, occupation, body size, robot familiarity, gaming habits, *etc.*, and observed their scores over 10 trials. The data is provided in Figure 7 as learning curve plots.

The goal of the analysis was to identify any differences in learning curves between the sample groups if any significant difference. The null hypotheses are the same for all categories: *“There is no difference between sample-* X *and sample-* Y *in learning criteria* L *”*:. We investigate the list of samples with respect to the learning criteria where the null hypothesis is rejected with 10% significance level.

As seen in Figure 7, all groups in each category have a similar learning curve which is increasing steep until around trial 3, keeps increasing with a relatively fixed gradient between trial 3 and trial 7, and reaches a plateau after trial 7. We designate those regions as 1) steep learning, 2) steady learning and 3) plateau. There is no user background factor that can be identified as significantly positive or negative in the progress of learning from this perspective. This supports a hypothesis of “everyone can cooperate” - or more precisely “everyone can *learn* to cooperate” - with robots.

Investigating the results more closely, there seems to be a drop in the score as the trial number increases in the majority of subplots in Figure 7 around 8^*th*^. This reverse learning effect is discussed in more detail in Section 4.2. Note also that the physical tiredness parameter seems to be one where there is more of a difference between the two sample groups. Although none of the users stated that they experienced severe physical tiredness or fatigue (see the mean and standard deviation, and the number of users in the sample group “low”), those who have lower physical tiredness have more fluctuating scores in the plateau region.

For most of the background factors, the two sample groups have relatively similar start levels, whereas, for some background factors, one sample group has a higher start score (*gender, job category, height, arm length, programming background, past gaming habits, proximity to robots, feeling gradual easiness, communication feeling, beliefs on would do better and physical tiredness*). While the higher start score in *gender, height, programming background, past gaming habits, proximity to robots and communicate feelings* is also reflected in higher end-scores, the higher start score for the *job category, arm length, feeling gradual easiness, beliefs on would do better and physical tiredness* converges to the same end score as the other sample group. The sample groups divided by age start and end in relatively similar scores, yet the steep learning region in higher age groups are even steeper. Lastly, there are no significant differences between the sample groups of *current gaming habits, robot and robot arm familiarity, visual feedback screen usage and mental tiredness* for the start and the end scores. Further details on the quantitative results for each background factor towards the learning criteria based on the results of the t-tests, respective *p*-values and effect sizes are given in Section 4.2.

## 4 Discussion

The learning progress of the different sample groups towards the different learning criteria, and the main findings regarding the results of the t-tests, respective *p*-values and effect sizes, are given in Table 1.

**TABLE 1:** Satistics table across user background and learning criteria. In total 34 background variables gave significant results out of 144 t-tests.

Category	Learning criteria	$\bar{x}$	$\bar{y}$	*s*_*x*_	*s*_*y*_	*n*_*x*_	*n*_*y*_	t-score	*p*-value	Effect size (d ∧ g)
Programming background	highest	674.00	683.43	12.46	9.21	18	14	−2.46	0.02	Large (-0.86)
Programming background	avg_3	660.41	674.10	16.76	12.06	18	14	−2.68	0.01	Large (-0.94)
Physical Tiredness	avg_first_2	551.08	459.71	54.23	109.76	25	7	2.13	0.07	Large (1.32)
Past Gaming Habits	highest	671.25	682.25	12.26	9.96	12	20	−2.63	0.02	Large (-0.99)
Past Gaming Habits	avg_3	657.33	671.83	18.49	12.14	12	20	−2.42	0.03	Large (-0.93)
Height	avg_first_2	491.38	554.92	99.86	50.55	12	20	−2.05	0.06	Large (-0.8)
Gender	var_3	56.22	22.09	50.47	30.89	10	22	1.98	0.07	Large (0.82)
Gender	avg_first_2	473.10	557.45	98.81	49.63	10	22	−2.56	0.03	Large (-1.08)
Gender	delta_learning	167.55	96.23	89.71	52.90	10	22	2.34	0.04	Large (0.97)
Feeling Gradual Easiness	var_3	21.03	58.56	27.38	53.28	22	10	−2.10	0.06	Large (-0.89)
Communication Feeling	highest	669.25	683.45	12.45	8.03	12	20	−3.53	0.00	Large (-1.36)
Communication Feeling	avg_3	657.89	671.50	16.94	13.76	12	20	−2.36	0.03	Large (-0.88)
Believes that would do better	avg_3	674.28	661.67	11.64	16.95	12	20	2.49	0.02	Large (0.87)
Believes that would do better	avg_last_5	663.85	639.12	16.42	29.75	12	20	3.03	0.01	Large (1.03)
Believes that would do better	avg_last_2	667.62	638.80	21.71	36.73	12	20	2.79	0.01	Large (0.96)
Arm Length	avg_first_2	489.67	555.95	97.99	51.27	12	20	−2.17	0.05	Large (-0.85)
Arm Length	delta_learning	163.75	91.38	75.35	58.23	12	20	2.86	0.01	Large (1.07)
Age	trial_no_avg_reached	7.91	6.57	1.22	2.01	11	21	2.33	0.03	Large (0.8)
Age	delta_learning	155.77	99.00	73.64	66.58	11	21	2.14	0.05	Large (0.81)
Programming background	avg_last_5	639.51	659.81	29.74	21.65	18	14	−2.23	0.03	Medium (-0.78)
Programming background	avg_first_2	506.39	562.86	86.24	53.34	18	14	−2.27	0.03	Medium (-0.79)
Past Gaming Habits	avg_last_5	636.20	655.71	33.14	22.29	12	20	−1.81	0.09	Medium (-0.69)
Mental Tiredness	trial_no_avg_reached	6.44	7.62	1.67	1.93	16	16	−1.86	0.07	Medium (-0.66)
Job Category	highest	682.91	675.62	9.17	12.69	11	21	1.86	0.07	Medium (0.66)
Job Category	delta_learning	90.00	133.45	47.20	80.70	11	21	−1.92	0.06	Medium (-0.66)
Height	avg_3	659.92	670.28	14.87	16.06	12	20	−1.85	0.08	Medium (-0.67)
Height	delta_learning	153.71	97.40	87.68	55.16	12	20	2.00	0.06	Medium (0.77)
Gender	avg_3	658.43	670.02	15.42	15.55	10	22	−1.96	0.07	Medium (-0.75)
Communication Feeling	trial_no_avg_reached	6.17	7.55	2.21	1.47	12	20	−1.93	0.07	Medium (-0.74)
Believes that would do better	var_3	17.63	41.83	24.66	45.82	12	20	−1.94	0.06	Medium (-0.66)
Believes that would do better	avg_first_2	564.17	511.25	53.15	84.74	12	20	2.17	0.04	Medium (0.75)
Familiarity with Robots	highest	674.76	681.93	12.13	10.95	17	15	−1.76	0.09	Medium (-0.62)
Familiarity with Robots	avg_3	661.57	671.87	17.94	12.38	17	15	−1.91	0.07	Medium (-0.67)
Familiarity with Robots	avg_first_2	510.03	554.97	92.21	50.98	17	15	−1.73	0.10	Medium (-0.6)

Note that in Figure 7, we can see subtle decreases in the score around trial numbers 7,8 and 9. According to our observations and user feedback, this could come from several reasons. First, participants were new to the HRC control scheme and how to use both arms to control the robot. Therefore, the participants focused in their first trials (on average four trials) to get used how to control the robot and their own body motions and gestures, rather than trying to solve the task as quickly as possible. However, as user confidence increased (particularly in the learned plateau), more risk-taking behaviour was observed, and users asked for more speed and tried new approaches to accomplish the task faster. This behaviour was observed more in users with competitive traits and those who had acquaintances enrolled in the experiment.

**FIGURE 8:**
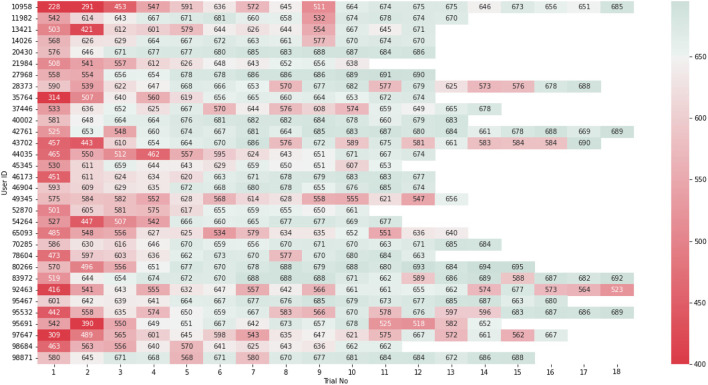
User score data on each trial. The results are colour-coded from red to green to visualize the training results from low to high.

Note also that the majority of the users requested to continue the experiment after the 10th trial and achieve even better performance. The data from these trials were not included in the analysis for fair analysis but can be seen in Figure 8.

### 4.1 Observed user behaviours

Relevant observations made by the supervisor during experiments are discussed in this section.

First, the user’s comfort with the experiment and the setting is important for performance. User comfort most likely differ between users who work in similar environments to where the experiment took place and those who had never been at campus. Observations on how the user felt after the first trial were noted during the experiments, and any discomfort can also be a compounding factor in the metrics related to feeling gradual easiness, communication quality, beliefs that would do better or worse. This will be discussed more in later sections.

A risk-taking behaviour as the user comfort increases can be seen in the measurements figure of the example user Figure 6. The 10th trial ends up with a lower score than 7-8-9^*th*^ trials. The reason was that the user learned how to smoothly transit from the APPROACH state to COLIFT state without any harsh grasping after trial number 5 (see the robot TCP line). However, in the 10th trial, the user was too quick lift the table before the robot’s grasp was completed, which violated the grasp action, and the robot did not start moving upwards which cost the user a few points. This type of behaviour was also seen for other participants as well as in different stages of the task such as elbow height adjustments, releasing before the goal post buttons were pressed, *etc.* Another observation linked to user learning was the change in the poking force magnitude. Two distinct behaviour was observed between users: 1) they would interact with higher forces because they thought of the robot as a sturdy machine, and 2) they would interact with lower forces because of fearing to damage the robot. As seen in the force (scale 0.005) line of Figure 6, this particular user started with a relatively high poking force (trials 1 and 2) but lowered it in the later trials (6,7,8 and 9). Even though the user experienced a problem in trial 10, she did not increase the magnitude of the interaction force–she had learned the necessary level of force required to transition into the next state.

Participants also implemented instructions differently. For instance, some users preferred using both hands equally in the APPROACH state, whereas some chose one hand (unintentionally, regardless of which was their dominant hand) and kept the other hand stationary.

In terms of requesting a speed change for the robot compared to the initial parameters as the trials went on, almost all users requested the robot move faster within the first 10 trials. In total, 3 people on APPROACH (all are after the 10*th* trial), 28 people on COLIFT (all are before the 10*th* trial) 28 people on RELEASE (all are before the 10*th* trial). Among those 3 states, the users preferred a faster speed as the robot leadership increased in the task. In the APPROACH state, where the human is the sole leader of the task, the users did not tend to request to go faster but they apparently were satisfied such that they did not want to reduce it either.

### 4.2 Background factors vs. learning criteria

Regardless of age, gender, job category, gaming background, and robot familiarity, the learning curves of all users are positively inclined in Figure 7. The user scores were evaluated in for 6 different learning criteria, and the significant participant background factors were calculated with a two-sample *t*-test as shown in Section 2.4. In total 34 [background parameter, learning criteria] pairs were found to be significant with a *p*-value less than 0.1 and effect size bigger than 0.5 as shown in Table 1.

We have avoided using a single learning criteria or single trial score to evaluate learning. Based on our observations during the experiments, some users changed behaviour after some trials which caused deviations and glitches in their score curves. The most common deviation was because of the “reverse learning” effect. Some users who experienced that they learned to use the system well in early trials pushed their limits to achieve a better high-score. Although participants were informed that there was no competition between users, some users with highly competitive traits prioritized a higher score over a consistent learning curve. On the other hand, other participants aimed to achieve the task with as little risk for failure as possible, and declined increasing the speed even though observations indicated that they could have managed this change well. Therefore, we have analyzed data using all 6 learning criteria towards the all background factors for the participants.

#### 4.2.1 User body size

**FIGURE 9:**
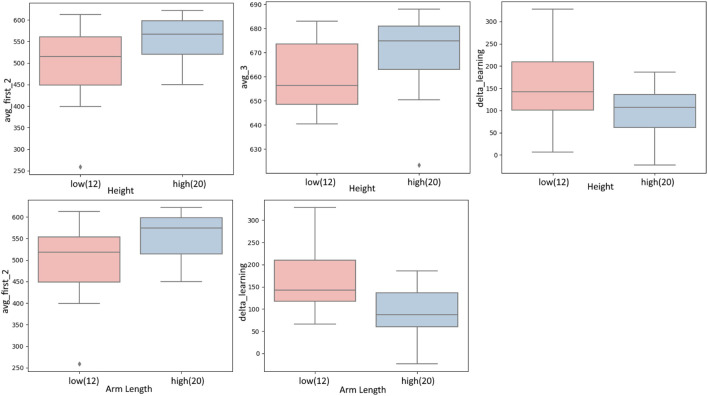
Body measures effect on learning. The pink group bar represents the lower measures and the blue group bar represents the higher measures of the respective numeric user factor (for this and the upcoming similar barplots).

The system is designed such that it does not require to be tuned between users to be easily integrated into industrial applications at a later stage. Since the human-to-robot motion mapping is a relative mapping, users with different body sizes should be equally able to use the system. From Figure 7, we see that the body size parameters *height* and *arm length* give similar progression of learning. However, according to Table 1 body size makes a difference in some learning criteria. Participants who are taller and have longer arms started with a better score (*avg_first_2*), while shorter participants showed a faster learning behaviour (*delta_learning*) as shown in Figure 9. In the end, there were no significant differences in the learning criteria except for the average of the best 3 scores *avg_3*. Note the effect of this user parameter is highly related to the model of the human and human-to-robot motion mapping method used in Section 2, and thus we cannot generalize that larger body sizes performs better. The takeaway from this experiment is that taller participants with longer arms performed better in *this* experiment, and that body size *could* play a role in HRC learning performance.

#### 4.2.2 Robot familiarity and anticipation

According to the *t*-test results in Table 1, users who had commercial robots at home or at work performed slightly better on some learning criteria than those who were not that familiar with robots. However, having a theoretical background in robotics does not seem to be significant for learning.The participants’ anticipation of the task had a bigger effect than assumed before the experiments. In total, 30 users out of 32 thought the first trials were hard, but that it got easier in later trials. Therefore, we took the mean of the given answers and applied this threshold to divide the participants into two sample groups. Those participants who felt continuous difficulty achieved more consistent scores than those who felt the operation became gradually easier. This could be related to higher risk-taking from users that felt the operation became gradually easier. Another explanation could be that participants who felt a gradual easiness performed worse in the start trials, and then performed much better in later trials. However, the *t*-test does not return any significant results either for *avg_first_2* or *avg_3*, which suggests that the risk-taking factor could play the bigger role.

Similarly, the experience of having established a good communication with the robot is relativeley high among all users; only one user partly disagrees with this. Therefore, we took the mean of the given answers and applied this threshold to divide the users into two sample gropus with different levels of communication with the robot. The feeling of good communication with the robot seems to have a positive impact in reaching higher scores (*highest* and *avg_3*), but it takes longer to reach the plateau of learning according to the *trial_no_avg_reached* learning criteria. Those who stated that they experienced a very good communication reached their consistent level of learning on average at the 8^*th*^ trial, whereas those who agreed or partly agreed with having established a good communication reached their level of learning on average at the 6^*th*^ trial. A possible reason for reaching the learning plateau slower could be that participants who commented positively towards having a good communication during the tests are observed to be more experimental and taking higher risks when they were about to reach the learning plateau.

In all (13/32) people expected to perform better, (14/32) people expected to perform worse (2/32) did not give any opinions, and (3/32) provided conflicting opinions (i.e., agreeing or disagreeing to both questions). There seems to be an ambiguity in the self-reported success/failure beliefs due to conflicting answers. However, it is observed in the results of the *t*-test that those who believed they could have done better get significantly lower scores in 3 out of 6 learning criteria. Although *delta_learning* was not found to be significantly different for this background factor, both the *avg_first_2* and *avg_last_2* were significantly different. This is an indication that users learned what could be objectively characterized as a good performance during the training. The results show that people who need more training could identify themselves based only on their own performance.

**FIGURE 10:**
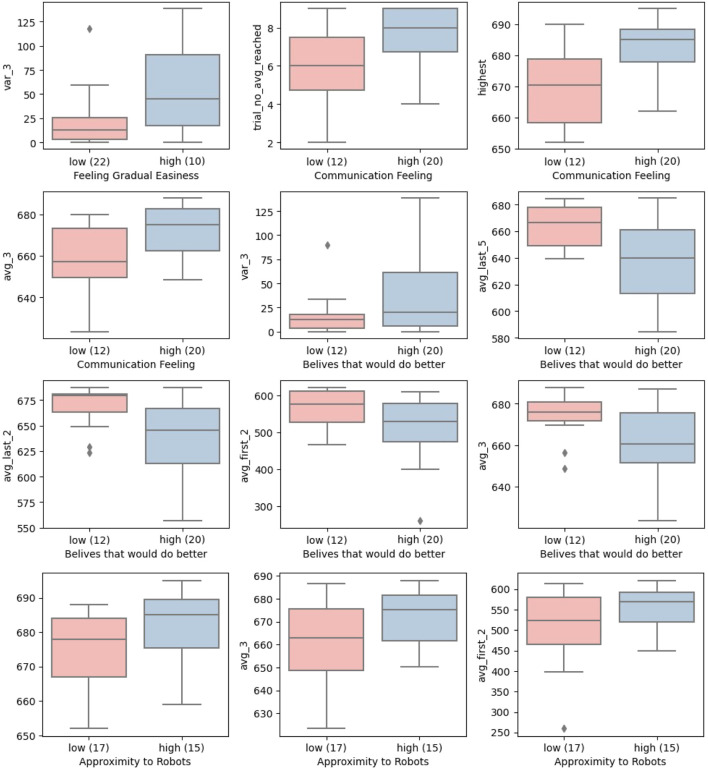
Robot familiarity and proximity effect on learning.

The overall comparison of the robot familiarity and anticipation with respect to different learning criteria is given in Figure 10.

#### 4.2.3 Age, gender and job category

The age parameter was significant for 2 different learning criteria, *trial_no_avg_reached* and *delta_learning*. Although the difference is not large, the higher age group seem to converge on a learning state faster than the younger age group. However, when it comes to the plateau of learning, younger participants achieved a higher plateau of learning compared to older participants. In all, age seems not to be a significant factor if enough training is provided.

Job category and gender should be looked at together because these two parameters are highly dependent on our group of participants. We registered people from 5 job categories; IT/engineering, health, craft, non-technical office job and unemployed. We analyzed data to see if IT/engineering-related jobs outperformed other jobs grouped together. Unfortunately, there were no women participants enrolled in IT/engineering jobs in the participant group, and thus the job category and the gender factor became indistinguishable in the learning criteria. From the analysis, we see that participants from IT/engineering jobs achieved significantly higher scores than other jobs. The amount of learning (*delta_learning*) among men was also lower compared to the amount of learning among women. Although the baseline (*avg_first_3*) of men were higher than women, and women learned more *delta_learning*, men seem to learn enough from their baseline so that the difference in the average of highest 3 scores *avg_3* is also significant in favour of men. Except for 3 outliers, men performed more consistently than women (See (*var_3*)).

**FIGURE 11:**
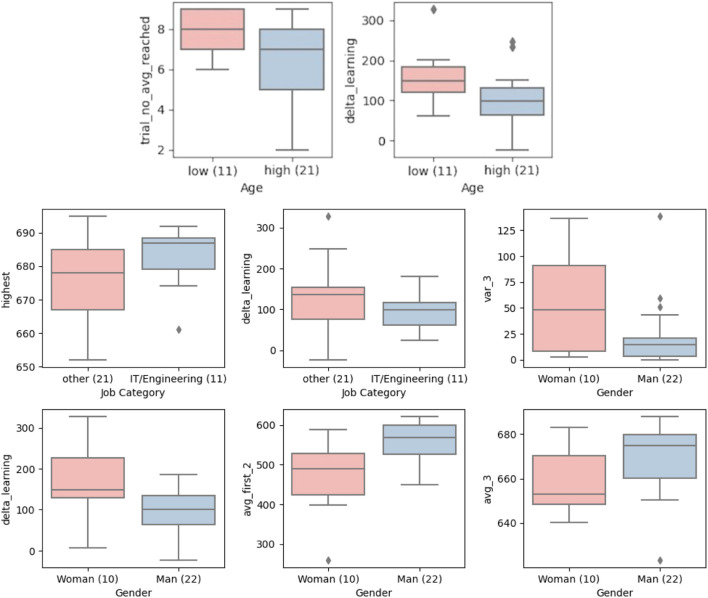
Job category and gender effect on learning. The blue group bar represents IT/Engineering related occupations in *Job Category* metric and male users in *Gender* metric. The pink and blue group bars represent other occupations registered such as teacher, craftsperson, nurse, consultant, unemployed, *etc.*, in *Job Category* metric and female users in *Gender* metric.

The overall comparison of the age, gender and job category with respect to different learning criteria is given in Figure 11.

#### 4.2.4 Gaming habits

**FIGURE 12:**
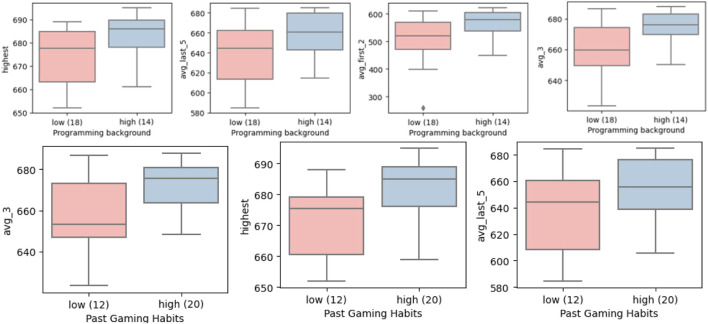
Programming skills and gaming habits effect on learning.

The programming background seems to be advantageous both for the baseline (*avg_first_2*) and for getting higher scores (*highest, avg_last_5* and *avg_3*) as shown in Figure 12. The current gaming habits do not seem to be a significant factor, which aligns with another relevant study in the literature (Tanaka et al., 2016), whereas past gaming habits seem to be advantageous in the same 3 learning criteria as for the programming background. We can note that the 3 users who were actively playing video games reported some confusion about the motion directions of the robot during the experiments. They reported that the mirrored motion of the robot was confusing since they were more used to a third-person or first-person view of controlling avatars. On the other hand, several users who did not have a lot of gaming experience reported the mirrored motion of the robot was intuitive, as the robot mimicked his/her motions. This should be taken into account when designing HRC systems that will be used by operators with different backgrounds in gaming.

#### 4.2.5 Physical and mental fatigue

**FIGURE 13:**
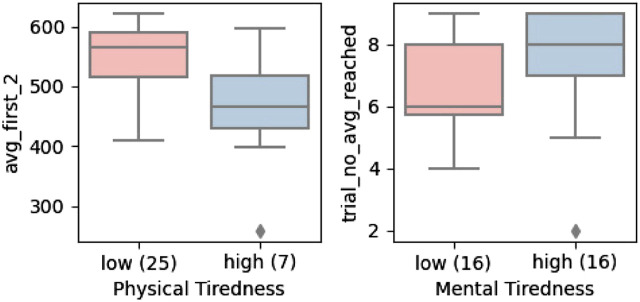
Mental and Physical tiredness effect on learning.

Neither mental nor physical tiredness was reported to be a challenging factor during the physical tasks as shown in Figure 7. Therefore, we took the mean of the given answers to the respective question and applied this threshold to divide the participants into two sample groups. The participants who experienced relatively low physical tiredness had a better start to the trials as shown in Figure 13, and the users who experienced relatively high mental tiredness seemed to reach the learning plateau slower.

### 4.3 Independence of user parameters and family-wise errors

As seen in the detailed comparisons, some parameters related to user background are not independent. For example, gender and job category. To analyse which parameters were dependent for our participant group, we ran the Chi-Square test and the resultant dependent parameters list is given in Table 2. This suggests that we should be very careful to draw any individual conclusions on the link between learning criteria and the background factors in Table 2 without acknowledging the possible dependency on other background factors.

**TABLE 2:** Chi-Square of independence: list of dependent parameters.

Category-1	Category-2	$X^{2}$	*p*
Gender	Job Category	5.56	0.02
Gender	Height	20.52	0.00
Gender	Arm Length	8.73	0.00
Height	Arm Length	14.22	0.00
Programming background	Gender	4.89	0.03
Programming background	Job Category	7.65	0.01
Programming background	Past Gaming Habits	7.62	0.01
Past Gaming Habits	Gender	4.69	0.03
Past Gaming Habits	Job Category	4.07	0.04
Familiarity with Robots	Gender	5.93	0.01
Familiarity with Robots	Job Category	6.22	0.01
Familiarity with Robots	Height	5.23	0.02
Familiarity with Robots	Programming background	12.43	0.00
Familiarity with Robots	Past Gaming Habits	9.11	0.00
Familiarity with Robots	Current Gaming Habits	4.07	0.04
Believes that would do worse	Mental Tiredness	4.66	0.03

In this study, we did not apply a family-wise error correction algorithm such as Bonferroni correction. There are two main reasons for that. First, there are debates on using Bonferroni correction Armstrong (2014) particularly when it is applied to the small sample sizes. Second, The family-wise error correction algorithms adjust the significance level to control the overall probability of a Type I error (false positive) for multiple hypothesis tests. Our null hypothesis is *two groups are equal* (_*H*_0: *μ*_1_ = *μ*_2_) and it is rejected when a significant difference is found between *μ*_1_ and *μ*_2_). Among 144 tests, we found only 34 that have an effect on learning considering the Type-I error is in this number. If a corrector was applied, this number would have been smaller yet the Type-II error would have been higher such that some background parameters which had an effect on learning would have been undetected. Our aim is to provide a suggestion list of user demographics that are more likely to have an effect on learning and they are better to be taken into account in further studies. At this point, it is more important to have more rejected *H*_0_s than it should be rather than accepting them wrongfully and missing some important background parameters that might have an effect on learning to cooperate with robots.

## 5 Conclusion and future work

This study investigates if anyone can learn to cooperate with robots through an experimental study with 32 participants performing a co-lift task, and which background factors such as age, gender, job, gaming habits, programming skills, familiarity with robots, *etc.*, impact learning. The co-lift experimental setup used IMUs for upper-body motion estimation and gesture recognition, and a gamified experimental setup to increase user motivation.

The results show that all users achieved a satisfactory level of cooperation with robots for the co-lift task regardless of background factors within seven or fewer trials. The rate of learning progression, level of achievement and how consistent the cooperation could be repeated for subsequent trials varied between different user groups, but the main conclusion is that all groups benefited from training irrespective of background factors and that all participants could achieve a satisfactory level of cooperation through training.

We believe that the results show that the focus for HRC systems developers should not only be on optimizing the technical setup for human-robot cooperation but also focus on better user training to increase HRC uptake in the industry. The results presented in this paper show that all users benefit from training to better cooperate with a robot and that achieving a satisfactory level of cooperation for any user irrespective of background can be done within a fairly short time and a limited number of training runs. This suggests that the user’s background is not the hindering factor when it comes to the adoption of HRC in the industry.

Finally, our study is limited to the co-lift task for HRC. Note that while the implementation of this task in this study was designed to be as generic and representative as possible for HRC tasks, we recognize that other HRC tasks that require other specific skills may give different results. However, we believe that the conclusions from this study apply to a wide range of HRC applications. Note also that the purpose of this study was not to reach a strict conclusion between user background factors and specific learning criteria, but rather to observe the learning process of the individuals and to investigate which background factors play a role in learning to better accommodate future implementation of HRC in industrial applications.

## Data Availability

The datasets presented in this study can be found in online repositories. The names of the repository/repositories and accession number(s) can be found below: https://doi.org/10.18710/CZGZVZ, DataverseNO, DRAFT VERSION.

## Footnotes

1
www.nsd.no/

2
https://youtu.be/_JZ-ENtvB7w

3 The data is currently under review of Open Access Guidelines at Western Norway University of Applied Sciences as of the date 19th of December, 2023.
